# Validation of cardiac output from Mostcare compared with a pulmonary artery catheter in septic patients

**DOI:** 10.1186/cc13330

**Published:** 2014-03-17

**Authors:** S Gopal, J Pooni, T Do, A Karimi, G Martinelli

**Affiliations:** 1 New Cross Hospital, Wolverhampton, UK; 2 University Hopsital of North Staffordshire NHS Trust, Stoke-on-Trent, UK

## Introduction

The Mostcare monitor is a non-invasive cardiac output monitor. It has been well validated in cardiac surgical patients but there is limited evidence on its use in patients with severe sepsis and septic shock [1].

## Methods

The first 22 consecutive patients with severe sepsis and septic shock in whom the floatation of a pulmonary artery catheter was deemed necessary to guide clinical management were included. Cardiac output measurements were simultaneously calculated and recorded from a thermodilution pulmonary artery catheter and from the Mostcare monitor respectively. The two methods of measuring cardiac output were compared by Bland-Altman statistics and linear regression analysis. A percentage error less than 30% was defined as acceptable for this study.

## Results

**Figure 1:**
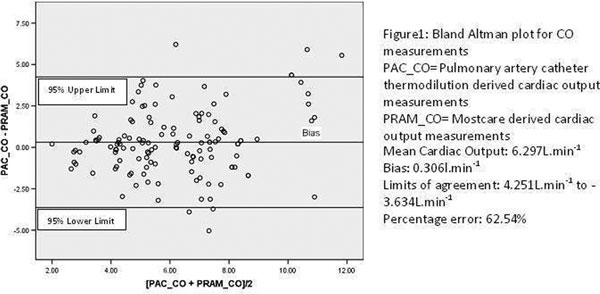
**Cardiac output Bland-Altman analysis**.

Bland-Altman analysis for cardiac output showed a bias of 0. 31 I/minute, precision (=SD) of 1.97 l/minute and a percentage error of 62.54%. Linear regression produced a correlation coefficient *r*^2 ^for cardiac output of 0.403. See Figure 1.

## Conclusion

Compared with thermodilution cardiac output, cardiac output studies obtained from the Mostcare monitor have an unacceptably high error rate. The Mostcare monitor is not a reliable monitoring device to measure cardiac output in patients with severe sepsis and septic shock on an ICU.
